# From Traditional to Targeted Immunotherapy in Myasthenia Gravis: Prospects for Research

**DOI:** 10.3389/fneur.2020.00981

**Published:** 2020-09-02

**Authors:** Renato Mantegazza, Carlo Antozzi

**Affiliations:** Neuroimmunology and Neuromuscular Diseases Unit, Fondazione IRCCS Istituto Neurologico “Carlo Besta”, Milan, Italy

**Keywords:** myasthenia gravis, autoimmunity, monoclonal antibodies, complement, clinical trials, Rituximab, Eculizumab, Fc receptor

## Abstract

Treatment of Myasthenia Gravis (MG) is still based on non-specific immunosuppression. Long-term high dose corticosteroids is still a major cause of side effects, in young as well as in elderly patients in whom comorbidities further increase the burden of chronic immunosuppression. Moreover, awareness of the limits of traditional therapies has led to the concept of “refractory MG.” The therapeutic approach to MG is therefore progressively evolving from the classic combination of corticosteroids and immunosuppressive drugs to new biological compounds targeting different immunopathological steps. Killing of B cells with Rituximab has been proposed and tested with positive results, particularly in patients with MuSK-associated MG. Therapeutic monoclonals against B cells at different stages of their maturation, or against molecules involved in B cell activation and function, represent a new area for further investigation. A differently targeted approach involved Eculizumab, a monoclonal antibody preventing the formation of C59b-induced MAC causing destruction of the neuromuscular junction. Data from clinical trials led to the approval of Eculizumab in the United States and Europe for MG. Since Eculizumab is a complement-targeted therapy, its use is limited to anti-acetylcholine receptor-associated MG, since anti-MuSK antibodies belong to IgG4 subclass and do not fix complement. Several anti-complement compounds are under investigation. An even more recent approach is the interference with the neonatal Fc receptor leading to a rapid reduction of circulating IgGs and hence of specific autoantibodies, an approach suitable for both anti-acetylcholine- and MuSK-associated MG. The investigation of compounds that selectively target the immune system will stimulate the search for specific biomarkers of disease activity and response to treatment, setting the basis for personalized medicine in MG.

## Introduction

Myasthenia gravis (MG) is an autoimmune disease of the neuromuscular junction (NMJ) characterized by weakness and fatigability of voluntary muscles (1). MG is a prototypical model of organ-specific autoimmunity in which target antigens and specific autoantibodies have been identified. The disease has been linked first to antibodies against the acetylcholine receptor (AChR), detectable in about 85% of patients, and more recently to the muscle-specific kinase (MuSK) or the lipoprotein-related peptide 4 (LRP4). MuSK and LRP4, together with agrin, are involved in NMJ formation and clustering of AChRs on the postsynaptic membrane. Specific autoantibodies impair neuromuscular transmission according to different mechanisms. Anti-AChR antibodies block the acetylcholine binding site of the AChR, increase internalization and degradation of AChRs and, since they belong to the IgG1 subclass, fix complement ultimately leading to destruction of the NMJ (2). Anti-MuSK antibodies belong mainly to the IgG 4 subclass and therefore do not activate complement, but impair neuromuscular transmission by interfering with agrin-related AChR clustering. Anti-LRP4 antibodies belong to the IgG1 subclass, activate complement, and interfere with the LRP4-agrin interaction pathway (2). Whatever the mechanism and antibody specificity involved, the final outcome is the impairment of neuromuscular transmission leading to the typical muscle weakness and fatigability complained by MG patients.

Therapy of MG, regardless of antibody specificity, is still based on symptomatic treatment and non-specific immunosuppression (3, 4). Cholinesterase inhibitors are the first-line treatment and maybe sufficient for mild MG at least at the beginning of the disease, but in the majority of patients variable degrees of immunosuppression are required and corticosteroids still represent the mainstay. Evidence of the efficacy of corticosteroids comes from retrospective studies spanning several decades showing that they are effective usually within a few weeks in generalized MG. The superiority of prednisone over placebo has been demonstrated by a randomized study in ocular MG; however the effect of corticosteroids in preventing generalization in ocular MG has not been demonstrated (5). Notwithstanding the proven rapid effectiveness of corticosteroids, the burden of long-term toxicity has been evident for many years, promoting the use of immunosuppressive drugs as add-on therapy with a steroid-sparing effect. Azathioprine and mycophenolate mofetil remain the most frequently used compounds, and demonstration of their clinical efficacy derives almost exclusively from retrospective studies. Indeed, end points of efficacy for mycophenolate mofetil were not reached in a randomized study, likely due to protocol design, and the drug is prescribed according to clinical experience (6, 7). Even the steroid-sparing effect attributed to non-biological immunosuppressive drugs has not been demonstrated in a controlled fashion except for azathioprine (8). A comprehensive review on immunosuppression in MG has been recently published (9). Cyclosporine and Tacrolimus, another inhibitor of calcineurin activity, but more potent than cyclosporine, are used as second-line therapy in MG patients, particularly in Eastern countries (10).

Immunomodulating therapies, i.e., those directly interfering with autoantibody activity such as intravenous immunoglobulin (IVIg) and plasmaexchange (PLEX), are used to obtain a rapid clinical response in patients with severe clinical compromise or in case of myasthenic crisis. IVIg and PLEX are considered equally effective according to results from randomized studies (11–13). The fast and short-term effect of PLEX is considered undisputable even though not investigated in a controlled fashion due to ethical reasons.

The occurrence of thymic abnormalities, particularly thymic hyperplasia reported in up to 70% of patients with early-onset MG, represents the immunopathological rationale for thymectomy as a therapeutic strategy to modify the natural course of the disease, with the idea of removing a site of autosensitization or perpetuation of the autoimmune attack (14). After four decades during which thymectomy was generally recommended for young-onset MG, a meta-analysis of the literature considered the procedure potentially capable of facilitating remission or improvement of MG, but still lacking a definitive demonstration (15). A controlled study published in 2016 showed that thymectomy improved the clinical outcome at 3 years and reduced the need for corticosteroids (16); remission was not recorded, but remission was not considered as an outcome in the study. Extension of the clinical observation up to 5 years still showed benefit from thymectomy and prednisone in non-thymomatous MG, albeit the patients' sample was small (17). At present, thymectomy is recommended for antiAChR-positive MG, increasingly performed with non-invasive techniques (18, 19). A further observation emerging from the above studies is that, even after thymectomy, MG still requires corticosteroids and immunosuppressive drugs for several years.

Our clinical experience in a very large series of MG patients treated according with traditional guidelines showed that complete stable remission was observed in 22% of AChR-positive MG patients, and about 30% were still symptomatic with various degrees of impairment at the end of the clinical follow-up (20). A shared clinical observation is that a subgroup of patients with MG can be affected with an unstable, poorly controlled form of the disease for a considerable time, leading to the concept of “refractory MG” (21–23). The definition of refractory MG is not a unique concept. The current definition includes patients failing to respond to adequate immunosuppression, or developing severe side effects or have comorbidities hindering the use of conventional therapies, patients needing frequent rescue treatment with IVIg or PLEX, or with frequent myasthenic crisis (24). Younger age at onset, female sex, history of thymoma, and positive MuSK antibodies have been associated with refractory MG in a series of patients (21). However, the burden of refractoriness goes far beyond the clinical features to which it has been associated and is likely to be considerably underestimated (25). Indeed, the impact on physical and mental functioning, ability to work and employment, and on activity of daily living need further investigation in order to be adequately weighed in the definition and assessment of refractoriness (24, 26). Moreover, we lack biomarkers correlated with response to treatments as well as guidelines on the optimal sequence of therapeutic interventions to adopt in refractory MG.

Despite the availability of several therapeutic options, the need to avoid the use of corticosteroids, or at least reduce their use as much as possible, is still unmet, and such a need is not limited to refractory patients but should concern all patients. Interestingly, RCTs in which the primary end-point was the reduction up to withdrawal of prednisone failed, though caveats in the protocols might have influenced the results (7, 27). Moreover, the effect of immunosuppressive drugs is usually too slow to justify their use as a single drug in the majority of patients, particularly in those with bulbar impairment. The duration of corticosteroid therapy in MG is not predictable, and in most patients spans from several months to years, not to mention patients who become steroid-dependent. A systematic analysis on the socio-economic impact of corticosteroids in MG is not available but the risk of health concerns including osteoporosis, metabolic, endocrine, ophthalmologic, and cardiovascular complications is considerable, even when corticosteroids are used in combination with immunosuppressive drugs. Another variable in the therapeutic decision is the increasing unwillingness to accept the iatrogenic burden of traditional treatments.

**Figure 1:**
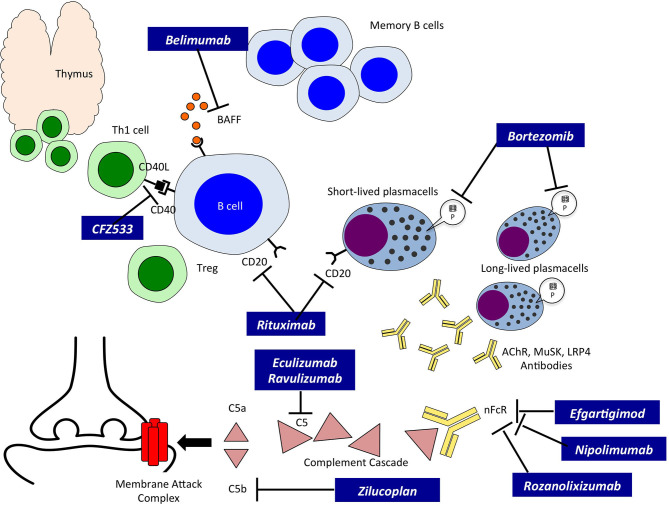
Innovative therapies in Myasthenia Gravis and their site of action. A schematic drawing of autoreactive B cells, T cells and Plasmablasts/Plasmacells leading to the production of autoreactive antibodies. The site of action of the new therapies, indicated in black boxes, is also indicated. BAFF, B cell activating factor; CD20, B-lymphocyte antigen CD20; CFZ533, monoclonal antibody to CD40; Th1, T helper cell type 1; Treg, regulatory T cell; P, Proteasome; AChR, Acetylcholine Receptor; MuSK, Muscle Specific Kinase; LRP4, low density lipoprotein receptor-related protein 4; nFcR, immunoglobulin neonatal Fc Receptor; C5, complement component C5; C5a and b, fragments of C5.

The introduction of new biological compounds directed specifically against different steps of the autoimmune process at the basis of MG has opened a new era in the field of its treatment. New classes of drugs, mainly biological, have entered clinical experimentation, and eventually reached Drug Agencies authorization; they belong to three major groups: a. Complement inhibitors; b. Neonatal Fc Receptor (nFcR) antagonists; and c. anti-B cell therapies (Figure 1).

### Complement Inhibitors

Among complement inhibitors, Eculizumab (ECU), a humanized monoclonal antibody, was the first drug tested due to its effect on complement-fixing anti-AChR antibodies, thus matching the concept of “Precision Medicine.” ECU targets C5 and prevents the formation of C5b which leads to the formation of the C5b-9 complex and thus prevents the effect of micro-destruction of the post-synaptic membrane, a crucial mechanism for the derangement of neuromuscular transmission (28). Clinical trials on ECU indicated that the drug was clinically effective, also in consideration that they included patients with refractory MG (29). Furthermore, ECU had a good safety profile as observed both in the Phase 3 study and the open-label extension (30). Notably, ECU safety in MG was similar to that observed in neuromyelitis optica (31) as well as in the long-term use for paroxysmal nocturnal hemoglobinuria (32). The risk of meningitis was negligible due to vaccination to Neisseria Meningitidis as to date only one non-fatal case was observed in a generalized MG patient concomitantly treated with two immunosuppressive drugs. Zilucoplan and Ravulizumab are other complement inhibitors currently tested in MG. Zilucoplan is a subcutaneously self-administered peptide of 15 aminoacids that binds specifically to C5 and prevents the cleavage of C5 into C5a and C5b; Zilucoplan gave positive results in a phase 2 study recently reported (33). Ravulizumab has been developed by re-engineering ECU to create a novel longer-acting antibody allowing administration every 8 weeks (34). Interestingly, Ravulizumab offers the opportunity of a subcutaneous administration hence allowing patients to be treated at home.

### Neonatal Fc Receptor Antagonists

Neonatal Fc Receptor (nFcR) antagonists is a new class of drugs used for the first time in MG. The capacity of these drugs to rapidly reduce circulating Igs offers a new therapeutic option for antibody-mediated disorders; if proven effective, nFcR will be an alternative to intravenous immunoglobulins or plasmaexchange, overcoming the increasing need of human plasma or the feasibility of apheresis when vascular access is poor.

nFcR antagonists include three groups of compounds: a) Recombinant Fc multimers, with multiple effects including FcRn targeting and inhibition of complement activation; b) Neonatal Fc receptor antagonists, including both IgG-derived Fc fragments, monoclonal antibodies or peptide mimetics; and c) antiFcgR antagonists. A comprehensive updated review on Fc-receptor targeting has been recently published (35).

Compounds under investigation in clinical trials in MG belong to nFcR antagonists, among these Efgartigimod, Rozanolixizumab, Nipocalimab (M281) and RVT-1401. The mechanism of action operates through the binding of the “antagonist” with the nFcR, a molecule responsible for IgG recycling at the endothelial level, and the binding results in a rapid and significant degradation (and reduction) of overall plasma IgG levels and hence pathogenic autoantibodies (36, 37). nFcR antagonists are very selective as they reduce IgG but not the other Ig isotypes or other plasma proteins, such as albumin. The clinical relevance of Efgartigimod, an engineered IgG1-derived Fc fragment, was given by the rapid (as early as 1 week) titer reduction of IgG associated with clinical improvement in MG-ADL, QMG, and MGQoL-15 scales (38); interestingly, the clinical improvement outlasted the recovery of IgG titer. Furthermore, the mechanism of action of nFcR antagonists enables treatment of both AChR- and MuSK-positive MG patients, since their mechanism of action is unrelated to complement activation. Rozanolixizumab, a humanized, high-affinity anti-nFcR monoclonal antibody administered subcutaneously provided promising results in a Phase 2 study (NCT03052751) on moderate to severe MG patients and is now tested in a Phase 3 double-blind, placebo-controlled, dose-selective (adaptive design) study. Nipocalimab, a fully humanized deglycosylated monoclonal antibody to nFcR is currently used in a Phase 2 study (NCT03896295) on moderate to severe MG patients. RVT-1401 (formerly IMVT-1401) is a human recombinant anti-nFcR monoclonal antibody under investigation in a phase 2 study in MG (NCT03863080).

Safety and tolerability of nFcR antagonists have been acceptable and different compounds share headache as the most frequent adverse event; infections were not different from those observed in the control groups considering severity and codification.

### Anti-B Cell Therapies

B cells are crucial elements in the immune pathogenesis of MG, hence drugs targeting selectively these cells are likely to be relevant for treatment. The relevance of B cells is intrinsic to the multiple roles played in immune responses, among them: (i) B cells act as antigen-presenting cells; (ii) B cells interact with follicular helper T cells to generate memory B cells; (iii) B cell maturation leads to plasmablasts and plasmacells which generate immunoglobulins, including autoantibodies [reviewed in (39)]. B cell-targeted therapies can be performed by molecules that attack B cells both directly and indirectly, or via cytokine blockade.

#### Direct B Cell-Targeting

Rituximab, a monoclonal antibody developed for the treatment of lymphoma, has attracted much attention in the treatment of MG as it targets CD20, a molecule expressed on B cells from the stage of pre-B cells to that of mature/memory B cells. Case series and non-controlled studies have reported a beneficial effect of Rituximab in MG, showing a class IV evidence, with a particular emphasis on MuSK MG patients (40, 41). A recent phase 2 RCT (NCT02110706) performed on MG patients receiving Rituximab as a steroid-sparing agent did not meet the primary end-point (a prednisone reduction of at least 30%) and the *in fieri* phase 3 study was halted because of futility. Another study (NCT02950155) is ongoing to evaluate, as primary end point, the percentage of patients with a QMG score ≤ 4 and a daily Prednisolone dose of ≤ 10 mg at 16 weeks after randomization to Rituximab or placebo. Interesting clinical data emerged from a systematic retrospective review of the literature with collection of information regarding 169 MG patients from different centers (42). The authors reported a greater proportion of positive outcomes for MuSK- as compared with AChR-positive patients, as well as a significant reduction in the number of patients who experienced a relapse. Univariate analysis showed that MuSK antibody status was the only factor associated with improvement after Rituximab treatment. Multivariate analysis confirmed the importance of MuSK antibody status; moreover, mild to moderate severity of MG and median age lower than 45 years at the time of treatment were predictive of a positive outcome. Reduction in antibody titer did not predict a positive response to Rituximab. A retrospective cohort study reported recently showed that patients treated early in the course of the disease showed a greater benefit in non-MuSK MG compared with conventional immunotherapies (43).

The use of Rituximab in randomized controlled trials and the post-marketing surveillance highlighted a number of adverse events with a wide range of severity. Rituximab appears to be well-tolerated with fewer side effects compared with those observed in more conventional therapies and chemotherapeutic regimens (42, 44). Rituximab use at present, however, should be carefully evaluated in the context of the benefit/risk ratio and the prospect of a chronic administration in the case of MG.

Several anti CD20 monoclonal antibodies are under investigation in several oncological diseases and Rheumatoid Arthritis and, hopefully potentially available for investigation in MG in the future (45, 46). Other anti-CD20 compounds include Ocrelizumab a recombinant, humanized anti-CD20 mAb that is approved for the treatment of primary progressive and relapsing multiple sclerosis, and ofatumumab, a cytolytic IgG1k fully human monoclonal antibody approved for the treatment of Chronic Lymphocytic Leukemia (47). Studies with these compounds in MG have not yet been proposed.

However, a limitation of Rituximab and similar compounds is that CD20 is not expressed on plasma cells and plasmablasts, the B cell subtypes responsible for antibody production. A new approach has been designed to target the B-cell maturation antigen (BCMA), a cell surface protein expressed only by antibody producing B cells, by means of CAR (chimeric antigen receptor) T cell technology. A phase Ib/IIa study to assess safety, tolerability and preliminary efficacy is ongoing in MG (Descartes-08, NCT04146051).

Another approach involved targeting of the CD40 signaling pathway, an approach that does not cause depletion of B cells but prevents their activation. Indeed, CD40 is expressed not only on B cells, but also on T cells and on antigen presenting cells. The binding of CD40L on T cell with CD40 on B cell leads to B cell activation and a cascade of events leading to differentiation into plasma cells and production of specific antibodies. CFZ533, a humanized monoclonal antibody against CD40, has been investigated in a RCT in MG; the results of the study are not yet available (NCT02565576) (48).

#### Indirect B Cell Targeting

Bortezomib is a dipeptide that, by binding the catalytic site of the 26S proteasome acts as a proteasome-inhibitor; it is registered for refractory or heavily treated multiple myeloma, and due to its pharmacological activity targets short and long lived plasmacells and, hence, could be potentially useful in MG. Bortezomib was effective in the treatment of EAMG (the experimental model of MG) and prevented the production of anti AChR antibodies by cultured thymic tissue (49, 50). A clinical study (NCT02102594) has been performed on antibody mediated autoimmune diseases, including MG, but no results have been posted yet. However, Bortezomib is associated with severe adverse events, e.g., 30% of treated patients showed a painful peripheral neuropathy, thus limiting their use.

Another interesting drug is Belimumab, a human monoclonal antibody that neutralizes BAFF, a B cell activating cytokine. Belimumab has been registered for treatment of systemic lupus erythematosus, an autoimmune disease with significant similarities with MG. Furthermore, elevated levels of BAFF were observed in MG patients (51). In the past years a Phase 2 RCT was conducted to evaluate clinical efficacy and safety of Belimumab: the primary endpoint was not met, but the study suffered several methodological flaws that prevented the assessment of a still potentially useful compound (NCT01480596) (27).

#### B Cell-Targeting via Cytokine Blockade

Interleukin 6 (IL-6) is a cytokine produced by several cell types including B cells, and is thought to be in involved in autoantibody production, making it a potential candidate for investigation in MG. Tocilizumab is an anti-IL-6-receptor humanized monoclonal antibody that binds to cell-surface and soluble IL-6 receptor and prevents the proinflammatory activity of IL-6. Indeed, Tocilizumab has been approved for treatment of Rheumatoid Arthritis. The drug has been investigated in Neuromyelitis Optica with promising results in preventing relapses (52). Anti-IL6 treatment reduced specific antibodies and improved signs of the disease in experimental MG (53). No studies have been performed yet, but preliminary evidence of its efficacy in two patients with refractory MG has been reported (54).

## Unanswered Medical Questions?

**Figure 2:**
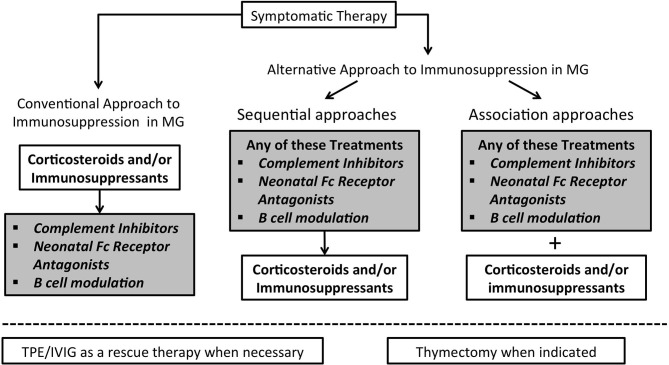
Algorithm for immunosuppression in Myasthenia Gravis may change in the next future. In the left part the conventional therapeutic approach to immunosuppression is illustrated; on the right side two possible different approaches are represented.

Will these drugs modify our current treatment strategies? Treatment of MG is a step-by-step approach in which decisions are based on the degree of clinical disability, taking into account comorbidities and the need to limit side effects. Such innovative therapeutics may significantly change our current approach to the treatment of MG and offer the opportunity to avoid, reduce or at least delay the use corticosteroids (45) (Figure 2). Most MG patients start with symptomatic treatment, but in a considerable proportion corticosteroids and/or immunosuppressants become necessary; IVIG and PLEX are used as rescue therapy in case of clinical deterioration. Indeed, whatever immunosuppressive drugs are employed, they are used on a chronic schedule that enhances the rate of adverse events, this being particularly true for corticosteroids. With the emergence of new therapeutic possibilities and rising reluctance of patients to accept the iatrogenic burden of traditional treatments, it remains to be seen whether patient compliance will improve.

The likelihood that doctors will prescribe innovative drugs will depend on: a. the ascertainment of their effectiveness as a first-line therapy and its ability to modify the course of the disease; b. the sustainability of the drug in clinical practice, particularly in the universalistic health systems; and c. the need to know the cost/effectiveness ratio for the disease treatment.

What data is still needed? At present, innovative drugs have been employed as add-on therapies and for most of them evidence of clinical benefit has been obtained. To date, a considerable time of follow-up (more than 3 years) is available only for Eculizumab. The length of follow-up and knowledge of long-term efficacy will be essential also for the other investigational products. We will need to know the ability of these drugs to work as immunosuppressants and how rapidly they can exert this effect; in this regard, we need to perform controlled clinical studies on MG patients naïve to immunosuppression. Such an approach is feasible with complement inhibitors and nFcR antagonist since they are fast in inducing clinical improvement (between 7 and 15 days), and the availability of rescue therapies should overcome ethical problems. Indeed, the time to obtain significant clinical benefit with both steroids and conventional immunosuppressants can be longer than that reported so far for complement inhibitors or nFcR antagonists.

## Future Directions

The introduction of compounds that target selectively the immune system will also offer a new opportunity to investigate immunological markers of disease activity and response to treatment. The topic of biomarkers in MG and other autoimmune disorders is not new but data available from series of patients treated with conventional therapies are, not surprisingly, still far from being conclusive and suitable for clinical application since too many immunological variables related to the disease and ongoing therapy are at stake simultaneously. The investigation of targeted therapies, due to their specificity, is likely to be more informative in the future, resetting the basis for personalized medicine in MG (45, 55).

In the past 3 years the horizon for improvement in immunosuppression has included different focused approaches which will hopefully address the unmet clinical needs of MG patients, as well as of patients affected with other autoantibody mediated diseases. If the expectations mentioned above will be met, a new era for the treatment of autoimmune diseases will be at hand and, prospectively, we could end up with a substantial modification on how to immunosuppress our MG patients, possibly with better results and improved quality of life.

## Author Contributions

RM and CA have equally conceived, drafted, and revised the manuscript. All authors contributed to the article and approved the submitted version.

## Conflict of Interest

RM received funding for research and congress participation from Sanofi-Genzyme, Teva, Bayer and BioMarin; participation in BioMarin, Alexion Pharmaceuticals and Argenx BVBA Scientific Advisory Boards. CA received funding for congress participation from BIOGEN.
